# Chlorhexidine–alcohol versus povidone–iodine as preoperative skin antisepsis for prevention of surgical site infection in cesarean delivery—a pilot randomized control trial

**DOI:** 10.1186/s13063-021-05490-4

**Published:** 2021-08-17

**Authors:** Athokpam Lenin Luwang, Pradip Kumar Saha, Minakshi Rohilla, Pooja Sikka, Lekha Saha, Vikas Gautam

**Affiliations:** 1 Department of Obstetrics and Gynecology, Postgraduate Institute of Medical Education and Research (PGIMER), Chandigarh, Pin 160012 India; 2 Department of Pharmacology, Postgraduate Institute of Medical Education and Research (PGIMER), Chandigarh, Pin 160012 India; 3 Department of Medical Microbiology, Postgraduate Institute of Medical Education and Research (PGIMER), Chandigarh, Pin 160012 India

**Keywords:** Antiseptic, Betadine, Chlorhexidine–alcohol, Cesarean section, Surgical site infection

## Abstract

**Objectives:**

To compare the efficacy of chlorhexidine–alcohol and povidone–iodine as preoperative antiseptic skin preparation for prevention of surgical site infection (SSI) after cesarean delivery (CD).

**Materials and methods:**

A total of 311 eligible women who underwent CS were recruited in the study after fulfilling all the eligibility and exclusion criteria. Patients were randomized into two groups (153 in chlorhexidine–alcohol group and 158 in povidone–iodine group) by a computer-generated randomization table. Patients were followed for a period of 30 days in postoperative period to monitor for SSI.

**Results:**

The rate of SSI in the chlorhexidine–alcohol group is 5.4% and that of the povidone–iodine group is 8.6%. *E. coli*, *K. pneumoniae*, and *Acinetobacter baumannii* were the most common organisms isolated. *E. coli* was found in 9.5% of the total SSI cases.

**Conclusions:**

The study found that the patients who received chlorhexidine–alcohol as skin antiseptic had less chance of developing SSI than those who received povidone–iodine; however, it did not reach a statistical significance.

**Trial registration:**

Clinical Trials Registry of India CTRI/2018/05/014294. Registered on May 31, 2018

## Introduction

Surgical site infection (SSI) is the second most common cause of nosocomial infections among the hospitalized patients covering about 14–16% of all nosocomial infections [1]. Post-cesarean complications due to infection have been estimated to occur in 7–20% of patients [2]. Globally, the average rate of CD is approximately 18.6% [3], being the most common major surgery performed among women.

The development of SSI after CD results in increased patient morbidity and increased duration of hospital stay due to infection, re-admission, use of healthcare resources, hospital costs, and burden on the mother and other family members or relatives and may also impair mother-child bonding and lactation [4, 5]. The rate of infection varies widely according to patient profile depending on several risk factors such as low socioeconomic status, maternal medical disorders, immunosuppression, steroid use, blood loss, body mass index, duration of surgery, duration of labor, rupture of membrane, absence of prophylaxis, and emergency CD [6–8].

There are many extrinsic factors attributing to SSI which include patient’s skin preparation, hand scrubbing techniques, environment of the operating room, processing of instruments, and hospital items which are to be used in the operating room [9]. Contamination of the surgical site by endogenous skin commensals or vaginal flora is a fundamental precursor to post-operative SSI after CD. Thus, infections are more of mixed polymicrobial which may include enterococci, gram-negative bacilli, group B streptococci, and anaerobes [10, 11].

Hence, choosing correct antiseptic for skin preparation becomes one of the crucial factors for prevention of SSI. Out of the different skin disinfectants available, povidone–iodine and chlorhexidine–alcohol are the most studied as they are active against gram-positive bacteria, gram-negative bacteria, virus, fungi, and *Mycobacterium tuberculosis* [12].

At present, there is no recommendation for a specific skin antiseptic preparation to be used before CD to prevent SSI. In the Cochrane Database of systemic review of 2018 on preoperative skin antiseptics for prevention of SSI after CD, it was found that chlorhexidine–alcohol was associated with lower rate of bacterial growth as compared to povidone–iodine, but the quality of evidence is very low [13].

Hence, this study was conducted to compare the antiseptic efficacy of chlorhexidine–alcohol and povidone–iodine so as to contribute in choosing the best antiseptic solution for preoperative skin preparation in CD.

## Materials and methods

This study was a pilot randomized controlled trial, conducted in the Department of Obstetrics & Gynaecology, PGIMER, from July 2016 to October 2017. The total number of deliveries during the study period was 8317. The total number of cesarean deliveries was 3207 (2179 emergency and 1028 elective cesarean deliveries). The study was approved by the Institutional Ethics Committee (ethical approval: No. INT/IEC/2017/561). The trial was registered by the Clinical Trials Registry of India (registration no. CTRI/2018/05/014294). Written informed consent was obtained from all study participants.

**Fig. 1:**
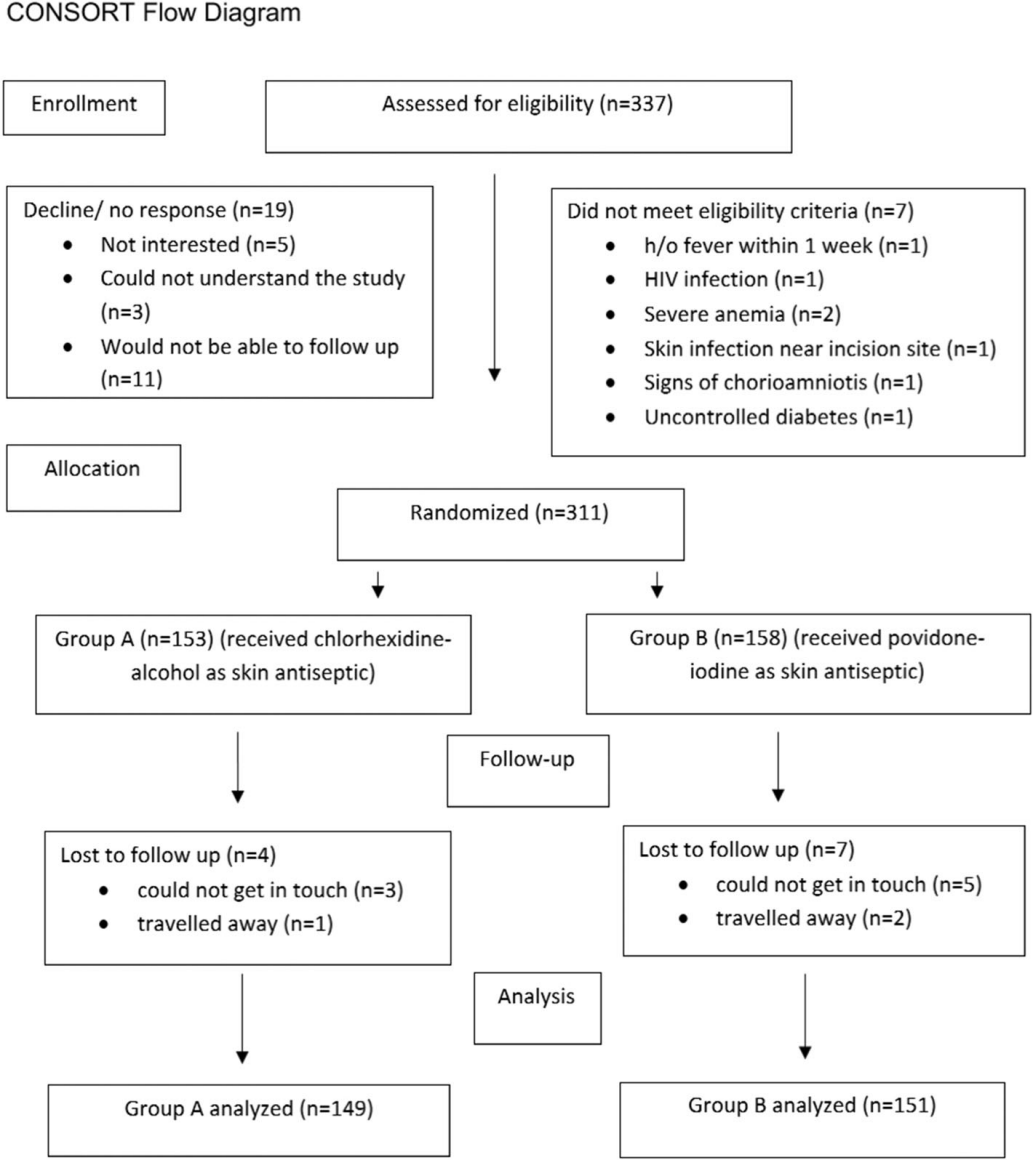
CONSORT flow diagram

A total number of 337 patients were assessed for eligibility. Randomization was done in 311 patients as 19 patients did not give consent for the study and 7 patients did not meet the eligibility criteria (Fig. 1). The present study followed the CONSORT 2010 guideline.

### Eligibility criteria

All pregnant women undergoing elective or emergency CD irrespective of gestational age at the Department of Obstetrics and Gynecology of the institute and consented for the study were included.

### Exclusion criteria

The exclusion criteria are as follows: patients with history of allergy to either of the disinfectants, any skin infection adjacent to the surgery site, severe anemia (Hb < 7gm/dl), h/o fever ≥ 38 °C on two or more occasions within 1 week before CD, pregnant women with features of chorioamnionitis, patients receiving immunosuppressants, heart disease, uncontrolled diabetes mellitus, and HIV infection.

Patients were randomized into two groups by a computer-generated random number table. Enrolment of the patient was done after the decision for CD had been made in case of emergency CD and 1 day prior to surgery for elective cases. Once a patient is enrolled, the antiseptic to be used is allotted as per the randomization table. The two groups are as follows:

Group A: Chlorhexidine–alcohol group (2% chlorhexidine–alcohol)

Group B: Povidone–iodine group (10% povidone–iodine)

Definition of SSI accepted for the study protocol was purulent discharge from the incision site, wound dehiscence, localized pain or tenderness, localized swelling, and erythema or heat within 30 days following CD [14]. A stitch abscess alone was not considered as a sign of SSI. The assessment of SSI was done by the faculty and was blinded to the group assigned.

Pubic hair clipping was done in all the patients. As per routine hospital protocol, prophylactic broad-spectrum antibiotic (cefazolin 2 g i.v.) was given 30–60 min prior to skin incision. As per study protocol, under strict aseptic precaution, a skin swab was taken from the surgical site before the application of antiseptic solution, and we labeled it as swab 1. Then, skin painting with antiseptic solution was started from the planned incision site with gentle pressure and proceeded to the periphery by widening circular motion. Once the desired boundaries or periphery was reached, the sponge was discarded and second painting was started, and the same procedure was repeated for 3 times [15]. A waiting period of 3 min was allowed after antiseptic application. As per study protocol, under strict aseptic precaution, a second skin swab was taken from the surgical site before incision, and we labeled it as swab 2. In all the cases, rectus sheath closure was done by PDS loop no.1 (polydioxanone monofilament, delayed absorbable, company ETHICON Inc.), and skin closure was done by mattress suture with Ethilon 2-0 (monofilament polyamide black, nonabsorbable, company Johnson and Johnson Pvt Ltd.).

The skin swabs were sent for culture and sensitivity test to the Department of Microbiology, PGIMER. The microbiologists are blinded about the antiseptic used for each patient.

Details of CD such as emergency or elective CD, type of skin incision, duration of surgery, amount of blood loss, and need for blood transfusion were recorded. Patients were examined for signs of SSI until discharge from the hospital. As per routine protocol, dressing was done after 48 h of operation. After discharge, they were followed up either by telephonic contact or outpatient department visit up to 30 days post-operative period.

For patient developing SSI, a wound swab (swab 3) was collected and sent for culture and sensitivity test.

Statistical analysis was conducted using IBM SPSS STATISTICS (version 22.0). Sample size was estimated based on previous study [16] considering the overall rate of SSI was significantly lower in the chlorhexidine–alcohol group than in the povidone–iodine group (9.5% vs. 16.1%; *P* = 0.004); our sample size came out to be 269 subjects at a power of 80% and confidence interval of 95%. For possible dropouts, it was decided to increase by 10%, so the final sample size is around 300 subjects in 2 groups. Proportions were compared using chi-square or Fisher’s exact test, depending on their applicability for the 2 groups.

The outcome measures of the study were as follows:

Primary outcome: Rate of SSI in both the study groups.

Secondary outcome: Organism growth on the swabs taken (swabs 1, 2, and 3).

## Result

A total of 311 patients were recruited in the study, out of which 11 were lost to follow-up. So, outcome analysis was done on 300 patients (Fig. 1).

In this study, we compared the efficacy of chlorhexidine–alcohol and povidone–iodine as skin antiseptic in prevention of SSI after CD. The baseline characteristics of the patients in both the groups are comparable such as age of the patients, period of gestations, BMI, and level of hemoglobin (Table 1). The surgical characteristics in both the groups are comparable such as types of surgery whether elective or emergency, types of anesthesia, types of incision, duration of surgery, amount of blood loss, and need for blood transfusion (Table 2).

**Table 1:** Baseline characteristics of study subjects in both the groups

Characteristics	Group A (*N*-149)	Group B (*N*-151)
Age in years (mean ± SD)	28.17 ± 4.75	27.85 ± 4.15
Period of gestation in weeks (mean ± SD)	36.68 ± 2.65	37 ± 2.49
BMI (mean ± SD)	25.03 ± 4.14	25.48 ± 4.28
Hb (mean ± SD) in gm%	11.48 ± 1.47	11.41 ± 1.37

Values are expressed as mean ± SD. BMI, body mass index, Hb, hemoglobin, PV, per vaginal, SD, standard deviation

**Table 2:** Surgical characteristics in both groups

		Group A (*N*- 149)	Group B (*N*-151)	Total	*p* value
Type of surgery	Elective	37 (24.8%)	16 (10.6%)	53 (17.7%)	0.572
	Emergency	112 (75.2%)	135 (89.4%)	247 (82.3%)	0.114
Type of Anesthesia	GA	2 (1.3%)	2 (1.3%)	4 (1.3%)	0.749
	SA	147 (98.7%)	149 (98.7%)	296 (98.7%)	0.104
Type of incision	Pfannenstiel	140 (94%)	144 (95.4%)	284 (94.7%)	0.284
	Vertical	9 (6%)	7 (4.6%)	16 (5.3%)	0.458
Duration of surgery in minutes (mean ± SD)		60.93 ± 14.75	59.40 ± 11.88	60.16 ± 13.38	0.321
Approx. blood loss (ml)		336.91 ± 112.16	363.58 ± 228.90	350.33 ± 180.81	0.202
Blood transfusion		4 (2.68%)	5 (3.31%)	9 (3%)	0.911

Values are expressed as mean ± SD or number (%). GA, general anesthesia, SA, spinal anesthesia, SD, standard deviation Grp, group. *P* value < 0.05—statistically significant

### Primary outcome

The overall rate of SSI is 7%, chlorhexidine–alcohol group had 5.4%, and povidone–iodine group had 8.6%, shown in Table 3. Statistical analysis for the test of significance using Pearson chi-square gives *p* = 0.276 which is statistically not significant. The relative risk is 0.624 and 95% confidence interval is 0.226 to 1.146.

**Table 3:** Rate and types of SSI according to CDC criteria [14]

Types of SSI	Antiseptic uses		Total (*N*-300)	*p* value	RR	95% CI
	CHA group, group A (*N*-149)	PVI group, group B (*N*-151)				
Total number of SSI (21)	8 (5.4%)	13 (8.6%)	21 (7%)	0.271	0.624	0.226 to 1.146
Superficial incisional	7 (87.5%)	12 (92.3%)	19 (90.5%)			
Deep incisional	1 (12.5%)	1 (7.7%)	2 (9.5%)			
Organ/space	0	0	0			
Number of days admitted in hospital (mean ± SD)	3.80 ± 2.47	3.76 ± 1.81				

Values are expressed as number (%)

CDC, Centers for Disease Control and Prevention

*P* value < 0.05—statistically significant

CHA, chlorhexidine–alcohol; PVI, povidone–iodine

RR, relative risk

CI, confidence interval

### Secondary outcome

Among swab 1, there were growth of *Enterococcus faecalis* (2 in group A and 1 in group B), *E. coli* (1 in group A) and *P. aeruginosa* (1 in group B). In swab 2, there was no growth of organism (Table 4). Among swab 3, there were growth of *E. coli* in 2 patients in group A and *K. pneumoniae* and *Acinetobacter baumannii* in group B (Table 5).

**Table 4:** Organism growth on swabs 1 and 2 in both groups

Growth of organisms	Group A		Group B		Total
	Swab 1	Swab 2	Swab 1	Swab 2	
*Enterococcus faecalis*	2	0	1	0	3
*E. coli*	1	0	0	0	1
*P. aeruginosa*	0	0	1	0	1
	3	0	2	0	5

**Table 5:** Organism growth on swab 3 in both groups

Growth of organisms	Group A	Group B	Total
*E. coli*	2	0	2 (9.5%)
*K. pneumoniae*	0	1	1 (4.7%)
*Acinetobacter baumannii*	0	1	1 (4.7%)
Sterile	6	12	18 (85.71%)
Total SSI (21)	8	13	21 (7%)

## Discussion

In this study, 7% (21 patients) of the study population developed SSI. In the chlorhexidine–alcohol group, the rate of SSI was 5.4% (8 patients), and in the povidone–iodine group, the rate of SSI was 8.6% (13 patients).

The rate of SSI in the study is close to that of the randomized controlled trial by Tuuli MG et al. [10] which was 4% in the chlorhexidine–alcohol group and 7.3% in the povidone–iodine group (*p* = 0.02). The study population is pregnant women undergoing CD. The sample size of this study is larger (*n* = 1147) than the present study. However, the most common organism isolated was *S. aureus*. The present study is also nearly similar to a systemic review and meta-analysis by Noorani A et al. [17] in clean-contaminated surgery where the rate of SSI was 6.1% in chlorhexidine–alcohol group and 9.8% in povidone–iodine group. Although this study is not confined to CD only, the study population is large (*n* = 5031, combining 6 eligible studies), and it was statistically significant.

Some studies in non-gynecological clean contaminated surgeries have also shown a lower incidence of SSI in the chlorhexidine–alcohol group than in the povidone–iodine group. Darouiche RO et al. [16] conducted a randomized study in patients undergoing clean-contaminated surgery, in which the rate of SSI was 9.5% in the chlorhexidine group and 16.1% in the povidone–iodine group. The study population size is 849. In another randomized controlled study conducted by Srinivas A et al. [18] in patients undergoing clean contaminated upper abdominal surgeries, the rate of SSI was 10.8% in the chlorhexidine–gluconate group and 17.9% in the povidone–iodine group. However, it was statistically not significant. The study population size is small (*n* = 342) which is nearly similar to the present study (*n* = 300). The present study also found less chance of developing SSI in the chlorhexidine–alcohol group, and it was statistically not significant.

However, there are studies which show that the rate of SSI in both the chlorhexidine–alcohol group and povidone–iodine group was almost similar. In a retrospective cohort study by Menderes G et al. [19], the rate of SSI in CD were almost the same in both the chlorhexidine–alcohol and povidone–iodine groups being 5% and 5.8% respectively. A limitation of this study was that it is not a randomized control study. In another randomized trial conducted by Ngai IM et al. [20] in preoperative skin preparation before cesarean delivery, there was no significant difference in the rate of SSI in both the chlorhexidine–alcohol group and povidone–iodine groups being 4.5% and 4.6% respectively. The size of the population is 1404. However, the study population is women undergoing non-emergent CD.

The CAPICA trial, May 2017, found that the rate of SSI in the chlorhexidine–alcohol group and the PVI group was almost similar, i.e., 6.3% and 7% respectively. It concluded that PVI should still be considered as appropriate antiseptic for cesarean section [21].

In this study, organisms such as *Enterococcus faecalis* (2 in group A and 1 in group B), *E. coli* (1 in group A), and *P. aeruginosa* (1 in group B) are found in culture report of swab 1. However, the culture report of the swab 2 of these patients showed no growth of organism. This shows that chlorhexidine–alcohol is effective against *Enterococcus faecalis* and *E. coli.* It also shows that the routine use of povidone–iodine of skin preparation is effective.

The bacterial growth in swab 3 was *E. coli* (2 in group A), *K. pneumoniae*, and *Acinetobacter baumannii* (1 in group B as mixed growth). *E. coli* is the commonest bacteria responsible for SSI in this study being 9.5% of total SSI. This is similar to another study from India by Shahane V et al. [22] in which it was found that the commonest pathogen isolated in SSI was *E. coli* (31.25%) followed by *P. aeruginosa* (25%) and *S. aureus* (22%). The two patients whose wound swab showed growth of *E. coli* had no history of prolonged rupture of membrane and no history of multiple PV examination or positive urine cultures. Both patients underwent emergency CD for pathological cardiotocography and placenta previa respectively. SSI is diagnosed during stitch removal on days 7 and 8 respectively. Hence, the possibility of *E. coli* on culture could be from the ascending infection from genitourinary tract.

Most of the SSI (80.96%) developed after getting discharged from the hospital. They presented mostly with discharge from the wound, pain, and swelling of the wound associated with or without fever. There was no need for readmission in any of the cases. But there was increased number of hospital visits for dressing and regular follow-up in outpatient department. During hospital stay, 19.04% of the SSI cases were diagnosed. Only two (9.52%) patients needed prolonged hospital stay. The average duration of hospital stay is 3–4 days.

Nineteen patients (90.5%) developed superficial incisional SSI, and two patients (9.5%) developed deep incisional SSI (Table 3). There was no organ/space SSI in both the groups in this study. Resuturing was done for one patient (4.76%) with deep incisional SSI for which she needed prolonged hospital stay for 19 days. All other patients with SSI were healed by secondary intention.

Prolonged leakage of membrane and number of PV examination also affect the postoperative morbidity after cesarean section [6, 23, 24]. In our study, 2 patients who developed SSI had preterm premature rupture of membrane and the duration of rupture of membrane was > 18 h. Another 2 had term premature rupture of membrane with duration of membrane > 18 h. Thus, 4 patients (19.06%) of those who developed SSI had prolonged rupture of membrane. The mean number of PV examination in both the groups is similar.

The strength of the study is that it is a prospective randomized controlled trial in a tertiary care institute, and swabs from the incision site were taken before and after application of antiseptics. However, the limitation of the study is that it has a relatively small sample size.

## Conclusion

The study found that the patients who received chlorhexidine–alcohol as skin antiseptic had less chance of developing SSI than those who received povidone–iodine; however, it did not reach statistical significance.

Since it was a pilot study, we recommend to study in larger population.

## Data Availability

The authors confirm that the data supporting the findings of this study are available.
